# Natural killer cells and BNT162b2 mRNA vaccine reactogenicity and durability

**DOI:** 10.3389/fimmu.2023.1225025

**Published:** 2023-08-25

**Authors:** Elizabeth K. Graydon, Tonia L. Conner, Kim Dunham, Cara Olsen, Emilie Goguet, Si’Ana A. Coggins, Marana Rekedal, Emily Samuels, Belinda Jackson-Thompson, Matthew Moser, Alyssa Lindrose, Monique Hollis-Perry, Gregory Wang, Santina Maiolatesi, Yolanda Alcorta, Anatalio Reyes, Mimi Wong, Kathy Ramsey, Julian Davies, Edward Parmelee, Orlando Ortega, Mimi Sanchez, Sydney Moller, Jon Inglefield, David Tribble, Timothy Burgess, Robert O’Connell, Allison M. W. Malloy, Simon Pollett, Christopher C. Broder, Eric D. Laing, Stephen K. Anderson, Edward Mitre

**Affiliations:** ^1^ Department of Microbiology and Immunology, Uniformed Services University, Bethesda, MD, United States; ^2^ Henry M. Jackson Foundation for the Advancement of Military Medicine, Inc., Bethesda, MD, United States; ^3^ Frederick National Laboratory for Cancer Research, Frederick, MD, United States; ^4^ Department of Preventive Medicine & Biostatistics, Uniformed Services University, Bethesda, MD, United States; ^5^ Clinical Trials Center, Infectious Diseases Directorate, Naval Medical Research Center (NMRC), Silver Spring, MD, United States; ^6^ General Dynamics Information Technology, Silver Spring, MD, United States; ^7^ Infectious Disease Clinical Research Program, Department of Preventive Medicine & Biostatistics, Uniformed Services University, Bethesda, MD, United States; ^8^ Department of Pediatrics, Uniformed Services University, Bethesda, MD, United States

**Keywords:** NK cells, mRNA vaccine, vaccine side effects, reactogenicity, antibody durability, SARS-CoV-2 vaccine, COVID

## Abstract

**Introduction:**

Natural killer (NK) cells can both amplify and regulate immune responses to vaccination. Studies in humans and animals have observed NK cell activation within days after mRNA vaccination. In this study, we sought to determine if baseline NK cell frequencies, phenotype, or function correlate with antibody responses or inflammatory side effects induced by the Pfizer-BioNTech COVID-19 vaccine (BNT162b2).

**Methods:**

We analyzed serum and peripheral blood mononuclear cells (PBMCs) from 188 participants in the Prospective Assessment of SARS-CoV-2 Seroconversion study, an observational study evaluating immune responses in healthcare workers. Baseline serum samples and PBMCs were collected from all participants prior to any SARS-CoV-2 infection or vaccination. Spike-specific IgG antibodies were quantified at one and six months post-vaccination by microsphere-based multiplex immunoassay. NK cell frequencies and phenotypes were assessed on pre-vaccination PBMCs from all participants by multi-color flow cytometry, and on a subset of participants at time points after the 1^st^ and 2^nd^ doses of BNT162b2. Inflammatory side effects were assessed by structured symptom questionnaires, and baseline NK cell functionality was quantified by an *in vitro* killing assay on participants that reported high or low post-vaccination symptom scores.

**Results:**

Key observations include: 1) circulating NK cells exhibit evidence of activation in the week following vaccination, 2) individuals with high symptom scores after 1^st^ vaccination had higher pre-vaccination NK cytotoxicity indices, 3) high pre-vaccination NK cell numbers were associated with lower spike-specific IgG levels six months after two BNT162b2 doses, and 4) expression of the inhibitory marker NKG2A on immature NK cells was associated with higher antibody responses 1 and 6 months post-vaccination.

**Discussion:**

These results suggest that NK cell activation by BNT162b2 vaccination may contribute to vaccine-induced inflammatory symptoms and reduce durability of vaccine-induced antibody responses.

## Introduction

Although initial formulations of mRNA vaccines have been tested since 1995 (1), the Pfizer-BioNTech COVID-19 vaccine (BNT162b2) was the first United States Food and Drug Administration (FDA) approved mRNA vaccine. Phase III clinical trials demonstrated that BNT162b2 was safe, and highly efficacious in preventing COVID-19 (2). Subsequent studies have shown that while protection against infection with novel variants can be variable, the vaccine still confers significant protection against development of severe disease (3). As of February 2023, over 400 million BNT162b2 vaccinations have been administered in the United States (4). While the basic tenets by which mRNA vaccines work are fairly well characterized, there remain many unknowns regarding the precise factors which drive immunogenicity and reactogenicity of mRNA vaccines.

Reactogenicity to BNT162b2 vaccination is very common but does not occur in all individuals. According to the Centers for Disease Control and Prevention (CDC), 84.7% of 18-55 year olds reported at least one local injection site reaction, and 77.4% reported at least one systemic reaction within seven days of vaccination (5). In the Prospective Assessment of SARS-CoV-2 Seroconversion (PASS) cohort, generally healthy adults reported substantial heterogeneity with regards to both severity and duration of local and systemic symptoms after BNT162b2 vaccination (6). In addition, while most individuals develop detectable IgG antibodies to SARS-CoV-2 spike protein after BNT162b2 vaccination, there is a wide range in the levels of peak antibody titers (7, 8).

The role natural killer (NK) cells play in influencing BNT162b2 reactogenicity or immunogenicity has not been fully explored. Other studies have shown early activation of NK cells within days of mRNA vaccine uptake in both animal models and humans (9–12). NK cells make up 5-20% of peripheral blood lymphocytes and non-specifically recognize pathogens and danger signals through activating and inhibitory receptors (13). NK cell activation results in cytotoxicity and the release of inflammatory cytokines (13). These functions contribute to the inflammatory environment and can influence the adaptive immune response (13).

NK cells are well recognized as having the ability to both amplify and diminish adaptive immune responses elicited by vaccines (14). After vaccination NK cells can release cytokines and stimulate antigen-presenting cells, which enhances the adaptive response (14). On the other hand, NK cell cytolytic activity can serve to contain adaptive immunity by reducing the number of responding T cells, which can subsequently diminish T cell help to B cells and reduce the quantity and quality of antibodies produced (14–16). Additionally, the role of NK cell activation and pro-inflammatory molecule release in local and systemic adverse symptoms after mRNA vaccination is not known.

Moreover, there is substantial person-to-person variation in terms of frequency, phenotype, and function of NK cells at baseline (before vaccination) (17). Thus, in this study, we assessed whether pre-vaccination NK cell frequencies and function are associated with inflammatory side effects or the magnitude and duration of antibody responses induced by BNT162b2 vaccination. To accomplish this, we analyzed peripheral blood mononuclear cells (PBMCs) collected from participants in the PASS Study (18), a prospective observational cohort study evaluating SARS-CoV-2 specific immune responses in healthcare workers at Walter Reed National Military Medical Center (WRNMMC).

## Methods

### Study participants

**Figure 1:**
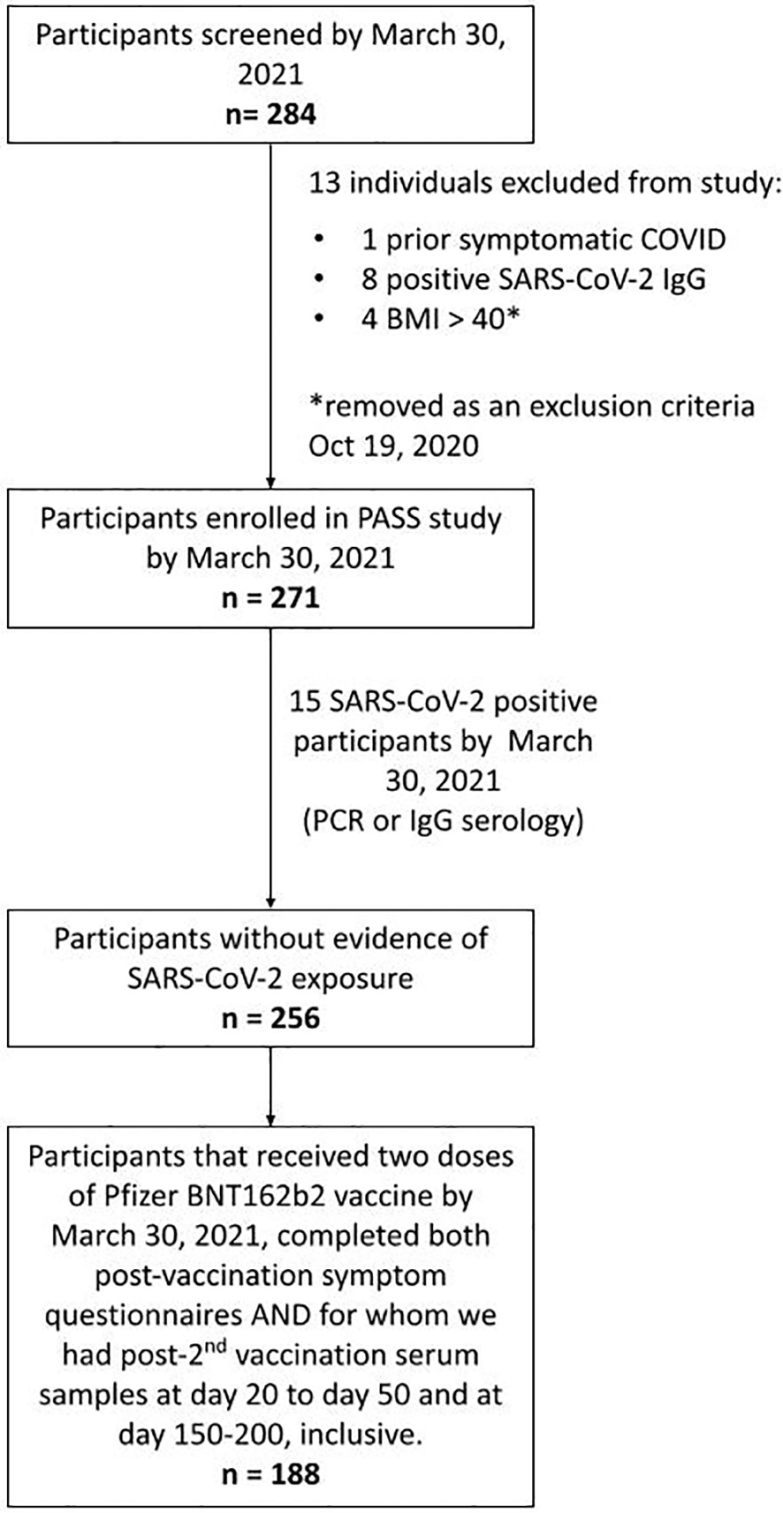
Strobe chart. Of the 271 individuals enrolled in the PASS study by March 30th, 2021, 15 were excluded due to evidence of SARS-CoV-2 exposure prior to vaccination. 188 of the remaining participants received 2 doses of the BNT162b2 mRNA COVID-19 vaccine by March 30th, 2021, completed symptom questionnaires following both vaccinations, and had serum samples obtained at time points between 20-50 and 150-200 days post-2^nd^ vaccination and comprised the final cohort used in this analysis.

Participants were enrolled in the PASS Study, an observational, longitudinal cohort study of healthcare workers (HCWs) that is evaluating clinical and immunological responses to SARS-CoV-2 infection and vaccination. The PASS study was initiated in August of 2020 with participants seen monthly during the first year of the study and then quarterly during the second year of the study at either the Naval Medical Research Center (NMRC) Clinical Trials Center or the Uniformed Services University (USU) Translational Medicine Unit. The study protocol was approved by the USU Institutional Review Board. Participants in the PASS study provided written informed consent to take part in the study. The cohort consists of generally healthy adults who are ≥18 years old, work at WRNMMC, are not severely immunocompromised, and were seronegative for SARS-CoV-2 at time of study enrollment (18). The subset of PASS participants included for analysis in this study also met the following criteria: 1) no history of COVID-19 diagnosis prior to vaccination, 2) remained seronegative for SARS-CoV-2 spike-specific IgG before vaccination during monthly testing, 3) received 2 vaccinations with the Pfizer/BioNTech BNT162b2 vaccine, 4) completed 2 vaccine-associated symptoms questionnaires by March 30, 2021, and 5) provided serum samples between 20-50 days and 150-200 days post-2^nd^ vaccination (**Figure 1**).

### Post-vaccine symptom assessment

Participants completed a structured vaccine-associated symptoms questionnaire at the first monthly visit after each vaccination dose as depicted in **Supplemental Figure 1**. Questionnaires asked about the presence and severity of 12 symptoms (8 categorized as systemic, 3 categorized as localized to the vaccine site, and 1 categorized as non-local and non-systemic). Severity of each symptom was defined as symptom intensity and measured on a scale of 0–4 (0 = not at all, 1 = a little bit, 2 = somewhat, 3 = quite a bit, 4 = a lot), with scores for each symptom summed for a total symptom severity score of 0–48.

### Antibody testing

Binding IgG antibodies against wild-type SARS-CoV-2 spike protein ectodomain trimers and receptor-binding domain (RBD) were measured using a microsphere-based multiplex immunoassay (MMIAs) built using Luminex xMAP-based technology as previously described (8). Anti-spike IgG levels were quantified in WHO binding antibody units (BAU) by interpolation against a pool of serum that had been calibrated against a US standard provided by Frederick National Laboratory. Anti-spike IgG BAUs have been shown to strongly correlate with serum neutralizing activity by both plaque reduction and pseudovirion neutralization assays.

### PBMC isolation and purification

PBMCs were isolated and cryopreserved from PASS participants at baseline and at various time points following COVID-19 vaccinations as previously described (18).

### Cell preparation and flow cytometry for NK cell phenotyping

Frozen PBMC samples were thawed and then washed with pre-warmed complete RPMI media (RPMI, 10% FBS, and 1% Penicillin/Streptomycin). Cells were resuspended in pre-warmed complete RPMI with 50 U/ml DNase (Invitrogen). Cells were then counted and transferred to FACS tubes at 1x10^6^ cells per tube in 100 µl cold 1X PBS. The viability compensation control was prepared by heat shocking cells at 70° C for 10min and mixing 1:1 with untreated cells. All cell samples, except for the unstained control sample, were then incubated with 1 µL of LIVE/DEAD™ Fixable Blue (Invitrogen) viability dye for 30 minutes on ice in the dark. Cells were then washed, resuspended in PBS/0.5% BSA, and incubated for 5 minutes with BD Horizon™ Brilliant Stain Buffer. Cells were then incubated for 30 minutes on ice in the dark with fluorochrome-conjugated antibodies to CD16 (BUV496), CD3 (BUV737), NKG2D (Super Bright 436), CD14 (BV510), CD19 (BV510), KIR3DL1 (BV711), CD57 (BV785), CD56 (FITC), KIR2DL2/L3/S2 (PE-Cy5.5), NKG2A (PE-Vio770), NKG2C (APC), and KIR2DL1 (APC-Vio770). The cells were washed, suspended in fixation buffer (BD Biosciences), and then incubated for 15 minutes at room temperature in the dark. After fixation, the cells were washed and resuspended in PBS/0.5% bovine serum albumin (BSA) and stored at 4° C until flow cytometry analysis. Single color reference controls were prepared using UltraComp eBeads™ compensation beads (Invitrogen). All antibodies were titrated prior to use to determine optimal staining concentrations. Fluorescence minus one (FMO) controls were used to establish positivity cut-offs for CD56, CD16, CD57, NKG2A, NKG2C, NKG2D, KIR2DL1, KIR2DL2/L3/S2, and KIR3DL1. All samples were acquired using a Cytek Aurora spectral cytometer (Cytek Biosciences) at the USU Biomedical Instrumentation Center Flow Cytometry facility, and the generated data was analyzed using the FlowJo Software v10 (BD Biosciences). Longitudinal samples from each individual were analyzed by flow cytometry on the same day.

The gating strategy to identify the NK cells and NK cell subsets is depicted in **Supplemental Figure 2**. Percent NK cells were calculated as the number of CD56+ NK cells (“Total NK Cells” gate) divided by the number of PBMCs (“Total Cells” gate) and then multiplied by 100. Absolute NK cell frequencies were calculated by multiplying the number of PBMCs/µl of blood by the percent NK cells previously calculated. Frequencies of the NK subsets were calculated as the number of the cells from the specific subset divided by the number of NK cells (“Total NK Cells” gate) and then multiplied by 100.

### NK cell functional assay

PBMC samples were thawed and rested overnight at 37°C in 5% CO_2_ in pre-warmed complete RPMI-1640 medium, supplemented with 10% FBS, 100U/mL penicillin, 100ug/mL streptomycin and 2mM L-glutamine (assay medium). The following morning, the PBMC were collected from wells of 6-well plates and counted. 50,000 PBMC were stained to assess the percentage of live CD3-/CD56+/CD16+ NK cells using LIVE/DEAD Fixable Near-IR Dead Cell Stain (Invitrogen) for 30 minutes at room temperature in the dark. Cells were washed twice and then incubated 30 minutes at 4°C in the dark with CD56 (PE), CD16 (PE), and CD3 (FITC). All samples were analyzed using a BD FACSCanto II instrument and the generated data analyzed using FCS Express (v6) (*De Novo* Software). A total of 1 x 10^6^ K562 target cells were labeled with 1µL Human TVA™ dye containing calcein-AM (Immunospot, CTL Inc.) in 1mL PBS for 20 minutes at 37°C. K562 cells were used because they express ligands that engage NK cell receptors that induce cytolysis (19). After this incubation and two washes in PBS, the K562 cells were resuspended at 1 x 10^5^/mL in assay medium. PBMC and K562 cells were then plated into a U bottom 96-well plate at NK cell: K562 (E:T) ratios of 10:1, 5:1, 2.5:1, and 1.25:1 in 200µL assay medium. Labeled K562 alone were also plated. The plate was centrifuged at 200xg for 1 minute and then incubated at 37°C in 5% CO_2_ for 4 hours. After the 4-hour incubation, each well was mixed and 50µL of the cell suspension was transferred to a flat bottom 96-well plate in triplicate. The plate images were acquired on a S6 Universal Analyzer (Immunospot, CTL Inc.), available at Frederick National Laboratory for Cancer Research, and live cell counts and the percent killing of target cells was determined using NK-Target cell Visualization Assay (NK-TVA) software as done by (20). To obtain a relative measure of the magnitude of highly cytotoxic NK cells for each individual, a NK cell cytotoxicity index was calculated by multiplying the percentage of target cells killed at specific E:T ratios by the absolute number of NK cells/µl of blood.

### Statistical analysis

Normality tests were run on each data set. If the data had a normal distribution, an unpaired t-test was used to compare two groups. If data were not normally distributed, a Mann-Whitney U test was performed for comparisons between unpaired groups. Spearman’s correlation was performed to evaluate relationships between factors, the correlation coefficient or Rho is given and is a nonparametric measurement of rank correlation. A one-way repeated measures ANOVA followed by Dunnett’s multiple comparisons test was used for comparisons between multiple time points. Statistical analyses were performed using GraphPad 9.

For the data showing percent killing or NK cytotoxicity index at multiple effector-to-target cell ratios, area under the curve (AUC) was calculated. AUCs were calculated in GraphPad 9 by calculating the area under each data point; those areas were then compared between each group and analyzed with either a t-test or Mann-Whitney U test depending on normality. Exploratory analyses of associations between NK cell receptor expression and symptom scores after 1^st^ and 2^nd^ vaccination, and IgG levels at 1 and 6 months post-vaccination, were performed using Spearman’s correlation followed by a Bonferroni adjustment for multiple comparisons

### Data availability

The data that support the findings of this study are available from the corresponding author upon reasonable request.

## Results

### Participant selection and demographics

By March 30^th^, 2021, the PASS study had enrolled 271 individuals. Fifteen of these participants were excluded from this study because they had evidence of SARS-CoV-2 infection before vaccination and the goal of this analysis was to evaluate the impact of NK cells on vaccine responses in SARS-CoV-2 naïve individuals. Of the remaining 256 participants, 188 completed symptom questionnaires after both 1^st^ and 2^nd^ vaccinations and had provided post-2^nd^ vaccination serum samples at time points between 20-50 and 150-200 days post-2^nd^ vaccination (**Figure 1**).

Participants self-reported demographic characteristics including sex, race, and ethnicity. Of the 188 individuals included in this study, 67.5% identified as female and 32.5% as male (**Table 1**). 171 individuals were Non-Hispanic, 12 were Hispanic, and 5 did not specify ethnicity. Participants identified their race as 72.3% White, 10.6% Black, 9.0% Asian, 2.1% another race, 5.3% multi-racial, and 0.7% did not specify their race. The average age was 42.4 years (range 20 - 69).

**Table 1:** Study participant demographics.

	All n/N (%)
Sex	
Female	127/188 (67.5)
Male	61/188 (32.5)
Ethnicity	
Non-Hispanic	171/188 (90.9)
Hispanic	12/188 (6.3)
Non-specified	5/188 (2.8)
Race	
White	136/188 (72.3)
Black	20/188 (10.6)
Asian	17/188 (9.0)
Other	4/188 (2.1)
Multiracial	10/188 (5.3)
Non-specified	1/188 (0.7)
Age	
Mean age (range)	42.4 (20–69)

### Evaluating the relationship between NK cell characteristics and sex or age

**Figure 2:**
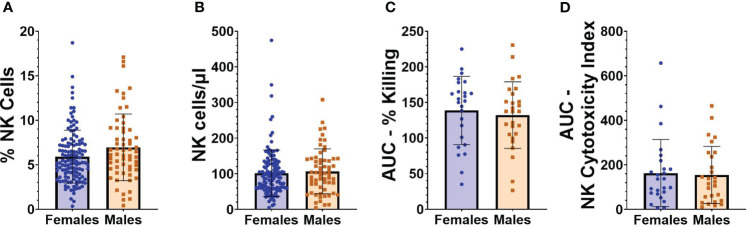
NK cell frequencies and functionality in relation to sex. **(A)** Percentage of NK cells from “total cells” gate, and **(B)** absolute number of NK cells/µl of blood in female (n=127) and male (n=61) participants at baseline (before vaccination). **(C)** AUC of percent killing of target cells by NK cells at effector to target (E:T) ratios 10:1, 5:1, 2.5:1, and 1.25:1 in female (n=25) and male (n=27) participants at baseline. **(D)** AUC of NK cytotoxicity index in female (n=25) and male (n=27) participants at baseline. Mann Whitney U test was performed for all comparisons between males and females.

Flow cytometry was performed on PBMCs collected from individuals at baseline, an average of 71.2 (SD 31.9) days before vaccination with BNT162b2. Given the relatively large size of our cohort, we initially evaluated whether NK cell frequencies, absolute numbers, or functionality were different in study participants based on either sex or age. As seen in **Figures 2A**, **B**, no statistically significant differences were observed between the frequencies or absolute numbers of NK cells in females compared to males (frequencies as a percentage of total cells: 5.9 [SD 2.9] vs 6.9 [SD 3.7]; absolute NK cell numbers/µl of blood 101.6 [SD 65.2] versus 106.9 [SD 63.0]). Functional cytotoxicity of NK cells was assessed on PBMCs from a subset of participants. No differences were found between AUCs of percent killing (**Figure 2C**) or NK cytotoxicity index (**Figure 2D**) in males versus females. Individual results at each E:T ratio for NK killing and cytotoxicity indices are shown in **Supplemental Figure 3**.

**Figure 3:**
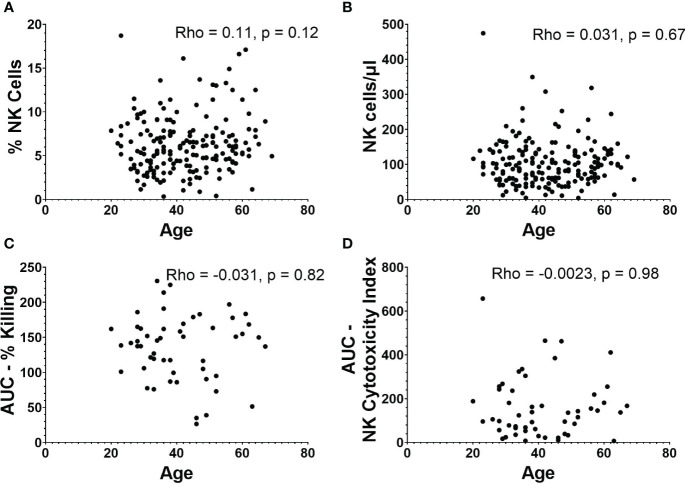
NK cell frequencies and functionality in relation to age. **(A)** Percentage and **(B)** absolute number of NK cells versus age (n=188). **(C)** AUC of percent killing of target cells by NK cells versus age (n=52). **(D)** AUC of NK cytotoxicity index versus age (n=52). Spearman’s correlation was performed to evaluate relationships of NK cells percentages, numbers, or killing with age.

There was no relationship between age and percentage of PBMCs that are NK cells (**Figure 3A**), or age and absolute NK cell numbers/µl of blood (**Figure 3B**). There was also no significant correlation between age and AUCs of NK cell functionality or NK cytotoxicity index (**Figures 3C, D**).

### Frequencies of NK cells at baseline, within one week after 1^st^ vaccine, and one month after 2^nd^ vaccine

**Figure 4:**
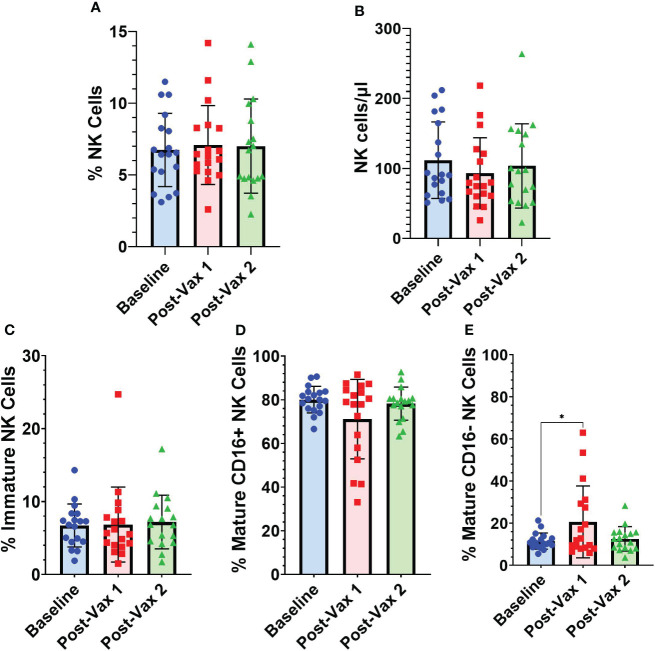
Longitudinal frequencies of NK cells. **(A)** Total NK cells (CD3^-^ CD14^-^ CD19^-^ CD56^+^) as a percentage of all PBMCs, **(B)** absolute NK cell numbers as NK cells/µl of blood, and NK cell subsets as a percentage of total NK cells: **(C)** immature NK cells (CD56^bright^ CD16^-^) **(D)** CD16^+^ mature NK cells (CD56^+^ CD16^+^), and **(E)** CD16^-^ mature NK cells (CD56^dim^ CD16^-^) at baseline (n=18), 1-7 days post-vaccine 1 (n=18) and less than 30 days post-vaccine 2 (n=17). Analyses between baseline and vaccination time points were conducted using one-way repeated measures ANOVA followed by Dunnett’s multiple comparisons test (*p<0.05).

A subset (n=18) of participants had PBMC samples obtained within one week of their first BNT162b2 vaccine dose. For these participants we conducted NK cell flow cytometry analyses at baseline, an average of 4.2 (range 1-7) days after 1^st^ BNT162b2 vaccine, and an average of 25 (range 14-41) days after 2^nd^ BNT162b2 vaccine to assess for any potential short-term changes in circulating NK cells following mRNA vaccination (**Figure 4**). Frequencies of total NK cells as a percentage of all PBMCs (**Figure 4A**), and absolute NK cell numbers/µl of blood (**Figure 4B**) did not differ significantly from baseline levels for the two post-vaccination time points we analyzed. While frequencies of immature CD56^bright^ CD16^-^ NK cells did not fluctuate substantially after vaccination (**Figure 4C**), frequencies of CD56^+^ CD16^+^ mature NK cells declined in some individuals during the first week after 1^st^ vaccination, but this difference was not statistically significant (**Figure 4D**). Interestingly, percentages of CD56^dim^ CD16^-^ mature NK cells were significantly higher during the first week after 1^st^ vaccination compared to baseline (11.51% [SD 3.8] vs 20.65% [SD 17]; p=0.02) (**Figure 4E**). As mature NK cells are known to lose CD16 expression upon activation (21), the decrease in CD16^+^ mature NK cells and commensurate increase in CD16^-^ mature NK cells suggests early NK cell activation after vaccination.

### Association between NK cell characteristics and symptoms after COVID-19 vaccination

**Figure 5:**
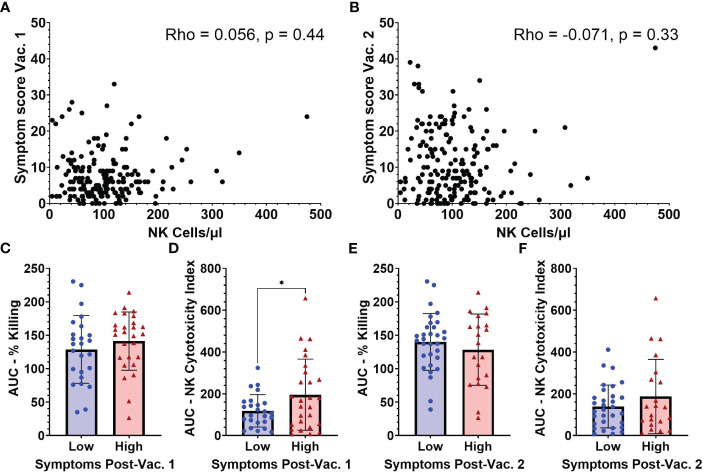
Symptom scores after vaccination in relation to NK cell frequencies and functionality measured at baseline (pre-vaccination). Absolute NK cells at baseline versus symptom scores following vaccination 1 **(A)** and vaccination 2 **(B)** (n=188). AUC for **(C)** percent killing of target cells by NK cells, and **(D)** NK cell cytotoxicity index in individuals with low (n=25) and high (n=27) symptom scores after vaccination 1. **(E)** Percent killing of target cells by NK cells, and **(F)** NK cell cytotoxicity index in individuals with low (n=31) and high (n=21) symptom scores after vaccination 2. Spearman’s correlation was performed to evaluate the relationship of NK cell percentages with symptom scores and unpaired t-test was performed for comparisons between low and high symptom score groups. *p < 0.05.

As NK cell activation by mRNA vaccination could potentially be an important contributor to vaccine-associated inflammatory symptoms, we compared baseline NK cell frequencies with symptom severity scores measured by structured questionnaires collected following both BNT162b2 vaccinations (**Supplemental Figure 1**). After the 1^st^ vaccination, participants reported an average symptom score of 7.45 (SD 6.19) and after the 2^nd^ vaccination an average symptom score of 11.0 (SD 9.22). The breakdown of the various local and systemic symptoms exhibited in the cohort is summarized in **Supplemental Tables 1**, **2**. As seen in **Figures 5A**, **B**, no significant associations were observed between absolute NK cell numbers/µl of blood and post-vaccination symptoms.

Due to constraints on conducting NK cell functionality studies on all samples, baseline functionality of NK cells based on target cell-killing was examined in subsets of individuals that had reported high (9 or higher) or low (3 or lower) symptom scores after the 1^st^ vaccine dose. After the 1^st^ vaccination, individuals in the low symptom score group had an average symptom score of 1.2 (range 0-3) while those with a high symptom score had an average symptom score of 16.8 (range 9-33) (data not shown). Following the 2^nd^ vaccination all individuals that had baseline NK functionality testing were regrouped into low and high 2^nd^ dose symptom score groups. Individuals in the low symptom score group after 2^nd^ dose had an average symptom score of 3.7 (range 0-9) while the high symptom score group had an average symptom score of 19.4 (range 11-43) (data not shown). The AUC for percentage of killing was not significantly different between the low and high symptom score groups after 1^st^ vaccination (**Figure 5C**). However, there was a significant difference between the baseline AUCs of the NK Cytotoxicity Index measured in low versus high symptom score groups after 1^st^ vaccination (118.5 [SD 77.5] vs 195.7 [SD 170.1], p=0.04) (**Figure 5D**). The higher NK Cytotoxicity Index values present before vaccination in individuals that became highly symptomatic after their first vaccine dose suggest that NK cells may be contributing to vaccine-related inflammatory symptoms.

Baseline NK percent killing (**Figure 5E**) and NK Cytotoxicity Indices (**Figure 5F**) were not statistically different between low versus high symptom groups after 2^nd^ vaccine dose. Individual results at each E:T ratio for NK killing and cytotoxicity indices after 1^st^ and 2^nd^ vaccination are shown in **Supplemental Figure 4**.

### Relationship between NK cell characteristics and spike-specific IgG levels after two BNT162b2 vaccinations

**Figure 6:**
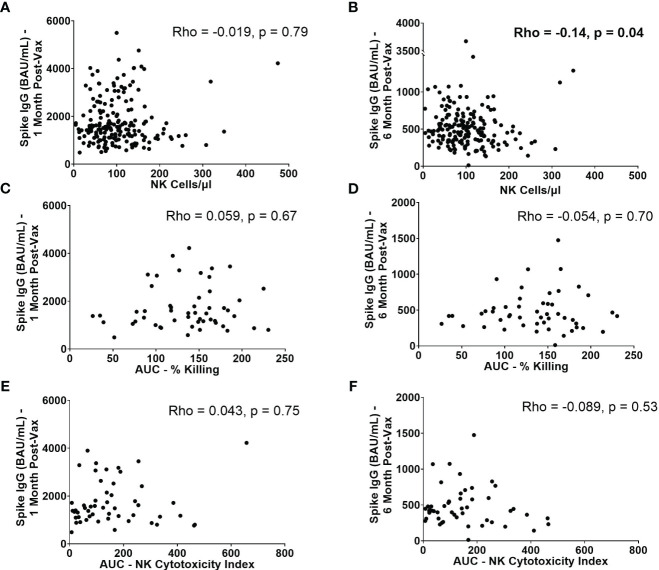
IgG levels at 1 month and 6 months post-2^nd^ vaccination in relation to NK cell frequencies and functionality. Absolute number of NK cells/µl of blood at baseline versus spike-specific IgG levels 1 month **(A)** and 6 months **(B)** post-2^nd^ vaccination (n=188). AUC of percent killing at baseline vs. spike-specific IgG at 1 month **(C)** and 6 months **(D)** post-2^nd^ vaccination (n=52). AUC of NK cytotoxicity index at baseline vs. spike-specific IgG at 1 month **(E)** and 6 months **(F)** post-2^nd^ vaccination (n=52). Spearman’s correlation was performed to evaluate the relationship of absolute number, or functionality of NK cells with spike-specific IgG levels at 1 or 6 months.

Because NK cells have the ability to abrogate or augment the development of adaptive immune responses, we next evaluated whether baseline NK cell frequencies and functionality are associated with higher or lower antibody levels post-vaccination. SARS-CoV-2 spike-specific IgG levels were measured from serum samples collected approximately 1 and 6 months after 2^nd^ BNT162b2 vaccination. While no statistically significant correlation was observed between absolute NK cell numbers pre-vaccination and spike-specific IgG levels at 1 month (Rho= -0.019, p=0.79) (**Figure 6A**), there was a statistically significant, weak negative correlation between absolute NK cell numbers pre-vaccination and spike-specific IgG levels at 6 months (Rho= -0.14, p=0.043) (**Figure 6B**). This finding suggests that high numbers of NK cells prior to vaccination may impede durability of mRNA vaccine-induced antibody responses. This negative correlation was also seen between the number of mature NK cells and spike-specific IgG levels at 6 months (Rho= -0.14, p=0.042) (**Supplemental Figure 5D**). There were no significant correlations between AUCs of NK cell killing or NK cytotoxicity indices or quantity of other NK cell subsets and IgG levels at 1 and 6 months post-vaccination (**Figures 6C–F**, **Supplemental Figure 5**).

### Immature NK cells expressing NKG2A are positively associated with IgG levels at 1 and 6 months post-vaccination

**Figure 7:**
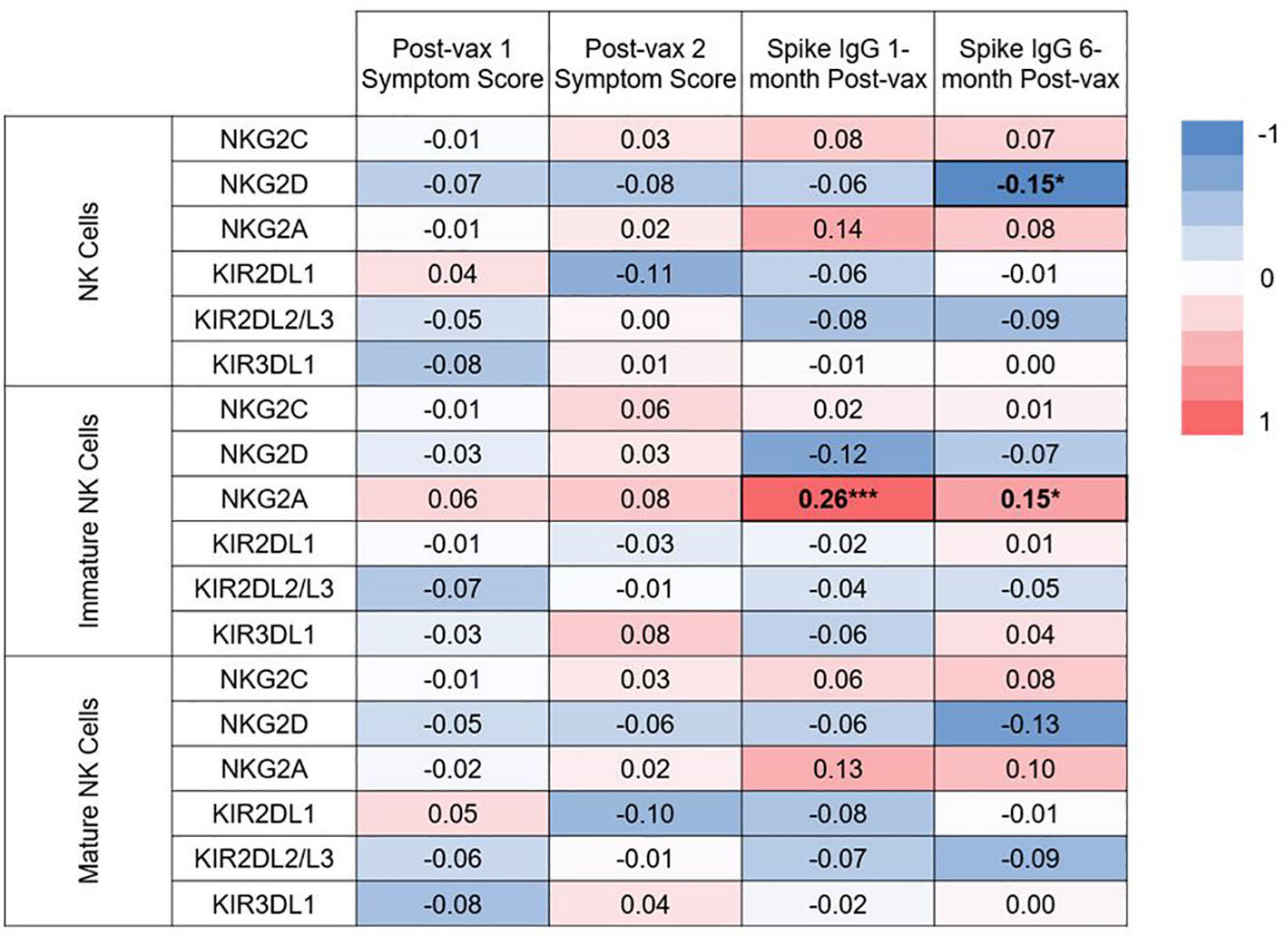
Heat map of correlations between baseline NK cell receptor expression and post-vaccination symptom scores, and IgG levels at 1 and 6 months post-vaccination. Spearman correlations were examined between expression of 2 activating NK cell receptors (NKG2C, NKG2D), and 4 inhibitory receptors (NKG2A, KIR2DL1, KIR2DL2/L3, KIR3DL1) on all NK cells, or expression on the immature or mature subsets on pre-vaccination samples. Spearman correlation rho values are given between expression of these receptors and post-vaccination symptoms scores and IgG at 1 and 6 months post-vaccination. *p<0.05, ***p<.001.

Functionality of NK cells are largely governed by their expression of activating and inhibitory receptors. We conducted secondary, exploratory analyses to assess for associations between expression of specific NK cell receptors and immunogenicity or reactogenicity. The associations between NK cell receptor expression pre-vaccination and symptom scores after 1^st^ and 2^nd^ vaccination, and IgG levels at 1 and 6 months post-vaccination are shown in **Figure 7**. Surface receptors examined included the NK activating receptors NKG2C and NKG2D, and the NK inhibitory receptors NKG2A, KIR2DL1, KIR2DL2/L3, and KIR3DL1. Receptor expression was assessed on total (CD3^-^ CD56^+^) NK cells, immature (CD56^bright^ CD16^-^) NK cells, and mature (CD56^+^ CD16^+^) NK cells. Frequencies with which each subset expressed individual markers are shown in **Supplemental Figure 6**. After conducting individual correlation analyses, negative correlation was found between expression of NKG2D on NK cells and spike-specific IgG levels 6 months post vaccination (rho= -0.15, p= 0.04). Positive correlations were found between NKG2A expression on immature NK cells and spike-specific IgG levels at 1 (rho= 0.26, p= 0.0003) and 6 months (rho= 0.15, p= 0.03) post-vaccination. NKG2A expression was also positively but not significantly correlated with higher spike-specific IgG levels at 1 and 6 months post-vaccination in total and mature NK cells as well. Interestingly, statistical significance remained for the positive association between NKG2A expression on immature NK cells and spike-specific IgG levels at 1 month post-vaccination even after applying a Bonferroni correction for 76 comparisons.

## Discussion

NK cells serve multiple functions within the innate immune system, with the ability to drive inflammation and also modulate inflammatory responses (22). Using a well characterized cohort of generally healthy adults with no evidence of SARS-CoV-2 exposure prior to vaccination, we were able to analyze for associations between pre-vaccination NK cell characteristics and BNT162b2-induced mRNA vaccine side effects and antibody levels. In this study, we observed that 1) circulating NK cells exhibit evidence of activation shortly following the 1^st^ vaccination with BNT162b2, 2) participants reporting higher symptom scores after the first BNT162b2 vaccination had a greater number of highly functional NK cells pre-vaccination compared to those who experienced little vaccine reactogenicity, 3) higher NK cell numbers correlated with diminished spike-specific IgG levels 6 months after completion of two vaccine doses, and 4) expression of the inhibitory marker NKG2A on immature NK cells was associated with higher antibody responses 1 and 6 months post-vaccination. These findings suggest that NK cell activation after BNT162b2 vaccination may contribute to vaccine-induced inflammatory symptoms and reduce durability of vaccine-induced antibody responses.

The finding that the mature CD16^-^ NK cell subset significantly increases following the 1^st^ BNT162b2 vaccination aligns with a study from Saresella et al. (12) which also observed increases in this NK cell population within a week of BNT162b2 vaccination. A previous study found that CD16 is cleaved when NK cells become activated (21). Therefore, the increase in the mature CD16^-^ NK cell population suggests that NK cells are becoming activated following vaccination with BNT162b2, which also aligns with previous studies (9, 11, 12, 23). In addition, data on the cleavage of CD16 indicates that CD16 expression on NK cells typically returns to 75% of baseline after 72 hours (21). We speculate that we observed increases in the mature CD16^-^ NK cell population after the 1^st^ vaccine dose but not after the 2^nd^ dose due to the time points analyzed (mean 4.2 days after 1^st^ dose versus mean 25 days after 2^nd^ dose).

The finding that people who reported high symptom scores after the 1^st^ BNT162b2 vaccine have significantly greater pre-vaccination NK cytotoxicity indices, compared to those with little reactogenicity, suggests that NK cells may be substantively contributing to the inflammatory processes causing these side-effects. Two other studies have examined the link between NK cells and post-BNT162b2 vaccination symptoms (23, 24). Syenina et al., found that individuals who experienced severe fatigue following BNT162b2 vaccination had elevated levels of expressed genes associated with NK cell activation post-vaccination (23), while Takano et al. did not observe an association with post-vaccine symptoms and NK cell activation (24).

High NK cell activity at the time of vaccination has been previously associated with weaker antibody responses in studies of yellow fever, malaria, and hepatitis B vaccines (25–27). Therefore, in this study we examined correlations between post-BNT162b2 antibody levels and baseline NK cell numbers, phenotypes, and functionality measurements. We found that while baseline numbers of NK cells were not associated with antibody levels one month after vaccination, they had a negative correlation with spike-specific IgG levels 6 months after two doses of BNT162b2. This finding indicates that NK cells might not have a major impact on the magnitude of peak antibody response induced by mRNA vaccination but may be important for mounting a durable immune response. Other studies have examined the correlation between NK cells and antibody levels in SARS-CoV-2 vaccination, with mixed results (11, 24). One found no correlation between baseline frequencies of NK cells and antibody titers at one month post-vaccination (11), aligning with the results of the present study. The second study utilized post-vaccination time points and found that individuals who had reduced NK cell numbers after vaccination produced greater titers of neutralizing antibodies (24).

To our knowledge, this is the first study analyzing baseline NK cell frequencies with longer term antibody time points after BNT162b2 vaccination. The finding that individuals with a greater number of NK cells have lower 6 month antibody responses is consistent with other investigations evaluating the role of NK cells in virus infection and vaccination. Specifically, studies have reported that activated NK cells after vaccination or viral infection can eliminate CD4 and CD8 T cells (28). For example, depletion of NK cells in mice infected with lymphocytic choriomeningitis virus results in markedly increased frequencies of virus-specific CD4 and CD8 T cells (29). NK cell depletion of activated CD4 cells not only has consequences for memory T cell responses but also has a negative impact on T follicular helper cell responses in the germinal center, which then affects vaccine antigen-specific antibody titers and plasma cells (14–16, 30). Evidence of this has been seen in mouse vaccination studies as well as in human vaccine clinical trials for malaria and hepatitis B (26, 27, 31). Consequently, because we observed lower antibody titers at 6 months post-vaccination in individuals with high baseline numbers of NK cells, we speculate that NK cells activated in response to BNT162b2 vaccination may be causing reduced antibody titers at late time points by reducing the T follicular helper cell response. Alternatively, it is also possible that high numbers of NK cells may be able to limit adaptive immune responses induced by mRNA vaccines by simply killing host cells expressing vaccine-delivered mRNA. We plan to investigate possible mechanisms of potential NK cell regulation of mRNA vaccine responses in future animal studies.

The data showing that NKG2A expression on immature NK cells is associated with higher levels of spike-specific IgG levels at 1 and 6 months post-vaccination requires further exploration. This finding was made as part of a large number of secondary analyses comparing expression of NK cell receptors with reactogenicity scores and antibody levels. Nonetheless, the result is intriguing. The NKG2A receptor recognizes HLA-E on cells and serves as one of the main inhibitory receptors for NK cells, serving to suppress their functions (32). We speculate that individuals with high levels of NKG2A expression may be predisposed to having less activation of NK cells after mRNA vaccination. If high numbers of activated NK cells suppress adaptive immune responses after mRNA vaccination, then it is possible that individuals predisposed to inhibition of NK cells would be more likely to develop high antibody responses to mRNA vaccine. With regards to other NK cell receptors, a similar study found a positive correlation between the baseline frequency of NKG2C+ NK cells and IgG levels at 1-month post-vaccination for SARS-CoV-2 (11). We did not observe this correlation with our cohort of individuals.

Of note, we also took advantage of our cohort to investigate whether NK cell numbers, phenotypes, or functional measures differ on the basis of sex or age. Numerous studies have compared NK cell frequency and function on the basis of these factors (33–48). Comparisons between sexes have yielded mixed results, with about half the studies reviewed concluding that males had greater numbers or frequencies of NK cells compared to females (35, 36, 42, 43, 45, 47), while the other half found no differences (34, 37, 38, 40, 48). In the literature, even more variability was found in functionality of NK cells between males versus females, but with no main conclusions drawn. Studies looking at age have found increases in total numbers of NK cells with age as well as a decline in functional activity (33, 49–52). Due to the relatively large size of our cohort, we analyzed for age- and sex-based differences to add to the current knowledge base in this area and to determine if we needed to consider these factors as possible confounders for our other analyses. We did not find any significant sex- or age-based differences in the absolute numbers or functionality of NK cells from our cohort. With regards to sex, while we observed a non-significant but greater frequency of NK cells in males, the absolute number of NK cells was almost identical between males and females. With regards to NK cell functionality, we suspect we did not observe age-related declines because our cohort was made up of generally healthy adults and we had no individuals over 69 years old.

The primary limitation of this study is that the main findings are based on correlational analyses. Therefore, we cannot be certain that increased reactogenicity and decreased antibody durability are definitively due to NK cell factors. Future studies could directly examine the impact of NK cells on vaccination in animal models. Another limitation is that for the longitudinal analyses there were only 18 individuals that had samples obtained within 1 week after 1^st^ vaccination and 1 month after 2^nd^ vaccination, and even for those there was variability in the number of days post-vaccination at which cells were collected. Other related papers observed NK cell activation within 3 days after administration of BNT162b2 (9, 24) and therefore we may have missed the peak response time of NK cell activation based on when samples were collected. Another limitation of the study is that we did not evaluate IFNγ  secretion nor markers of degranulation in the context of the target cell killing assay, and in the flow cytometry panel only evaluated a subset of known NK cell activating and inhibitory molecules.

Overall, the data suggests that NK cells may drive some of the reactogenicity experienced following mRNA vaccination, and that large numbers of NK cells may negatively affect the long-term durability of mRNA vaccine antibody responses. The extent to which NK cells influence mRNA vaccine responses, and whether this effect is specific for mRNA vaccines, will need to be addressed by future studies. Taken together, these results have important implications for efforts aimed at optimizing future mRNA vaccine designs. If future studies corroborate that NK cells play a role in reactogenicity and durability of immune responses induced by mRNA vaccines, then novel strategies could be considered to decrease engagement and activation of NK cells following vaccination.

## Data availability statement

The raw data supporting the conclusions of this article will be made available by the authors without undue reservation. Data for this study are available from the Infectious Disease Clinical Research Program, headquartered at the Uniformed Services University (USU), Department of Preventive Medicine and Biostatistics. Review by the USU Institutional Review Board is required for use of the data collected under this protocol. Data requests may be sent to: Address: 6270A Rockledge Drive, Suite 250, Bethesda, MD 20817. Email: contactus@idcrp.org.

## Ethics statement

The studies involving human participants were reviewed and approved by the Uniformed Services University (USU) Institutional Review Board (FWA 00001628; DoD Assurance P60001) in compliance with all applicable Federal regulations governing the protection of human participants. The patients/participants provided their written informed consent to participate in this study.

## Author contributions

Conceived and designed the study/experiments: EKG, CO, EG, BJ-T, AL, AM, SP, CCB, EDL, SKA, EM. Acquired data/performed experiments: EKG, TC, KD, EG, S’AAC, MR, ES, BJ-T, MM, MH-P, GW, SMa, YA, AR, MW, KR, SMo, JI. Created detailed analysis plan and/or analyzed the data: EKG, CO, EG, BJ-T, AL, AM, SP, CCB, EDL, SKA, EM. Interpreted findings: EKG, CO, SKA, EM. Contributed resources; reagents/materials/specimens: EKG, EG, S’AC, BJ-T, MM, MH-P, GW, SMa, YA, AR, MW, KR, JD, EP, OO. Composed first draft of manuscript: EKG, EM. Provided critical revisions and edits to provisional drafts: EKG, EG, AL, AM, SP, SKA, EM. Reviewed and approved final version for submission: (all authors) EKG, TC, KD, CO, EG, SC, MR, ES, BJ-T, MM, AL, MH-P, GW, KR, JD, EP, OO, MS, SMa, SMo, JI, DT, TB, RO’C, AM, SP, CCB, EDL, SKA, EM.

## Author disclaimer

The views expressed in this presentation are the sole responsibility of the presenter and do not necessarily reflect the views, opinions, or policies of the Uniformed Services University of the Health Sciences, the Department of Defense the Defense Health Agency, the Department of the Navy, Army, Air Force, the United States Government, or the Henry M. Jackson Foundation for the Advancement of Military Medicine, Inc. (HJF). Mention of trade names, commercial products, or organizations does not imply endorsement by the U.S. Government. The study protocol was approved by the USU Institutional Review Board in compliance with all applicable federal regulations governing the protection of human subjects.

## Supplementary material

Supplementary Figure 1 Symptoms questionnaire filled out by study participants after each SARS-CoV-2 vaccination. Participants of the PASS study answered these 12 questions following vaccination and selected on a scale from 0-4, where “0” means they did not experience the symptom at all and “4” means they very much so experienced the symptom. The total maximum score possible was 48 and the total lowest score possible was 0.

Supplementary Figure 2 Flow cytometry gating strategy for NK cell populations identification. First, a “Time” gate was drawn from a Time x FSC-A plot to capture events only at times when there was a steady flow of cells. Second, a “Singlets” gate was drawn using an FSC-A x FSC-H plot to exclude aggregated cells. Third, a “Live cells” gate was drawn using an FSC-A x Viability plot to exclude stained dead cells. Fourth, a “Total cells” gate was drawn using an FSC-A x SSC-A plot to capture the PBMCs (lymphocytes, monocytes, and granulocytes). Fifth, a “CD3, CD14, CD19 exclusion” gate was drawn using a CD14, CD19 x CD3 plot to select the cells that were negative for all 3 of these markers. Sixth, a “Total NK cells” gate was drawn using a CD3 x CD56 plot to select for all CD56^+^ cells (already gated as CD3^-^). Seventh, immature NK cell (CD56^bright^ CD16^-^), mature CD16^+^ NK cell (CD56^+^ CD16^+^), and mature CD16^-^ (CD56^dim^ CD16^-^) NK cell gates were drawn using a “NK Cell Subsets” CD16 x CD56 plot.

Supplementary Figure 3 Functionality of NK cells in female versus male participants. **(A)** Percent killing of target cells by NK cells at various effector to target (E:T) ratios in females and males at baseline. **(B)** NK cytotoxicity index, as determined by percent killing multiplied by absolute NK cell numbers, in females and males at baseline (n at 10:1, females = 23, males = 25; n at 5:1, 2.5:1, and n at 1.25:1, females = 25, males = 27). Mann Whitney U test was performed for all comparisons between males and females.

Supplementary Figure 4 Functionality of NK cells on participants with low versus high symptom scores after vaccination 1 and 2. **(A)** Percent killing of target cells by NK cells, and **(B)** NK cell cytotoxicity index, calculated as product of percent killing of target cells and absolute NK numbers, in participants with low and high symptom scores after vaccination 1 at E:T ratios of 10:1 (low n=23, high n=25) 5:1, 2.5:1, and 1.25:1 (low n= 25, high n=27). **(C)** Percent killing of target cells by NK cells, and **(D)** NK cell cytotoxicity index, calculated as product of percent killing of target cells and absolute NK numbers, in participants with low and high symptom scores after vaccination 2 at E:T ratios of 10:1 (low n=29, high n=) 5:1, 2.5:1, and 1.25:1 (low n= 31, high n=21). Unpaired t-tests were performed for comparisons between low and high symptom score groups. *p < 0.05.

Supplementary Figure 5 IgG levels at 1 month and 6 months post-2^nd^ vaccination in relation to NK cell subsets. Absolute number of immature **(A, B)**, mature CD16^+^ **(C, D)**, and mature CD16^-^ **(E, F)** NK cell subsets/µl of blood at baseline versus spike-specific IgG levels 1 month **(A, C, E)** and 6 months **(B, D, F)** post-2^nd^ vaccination (n=188).

Supplementary Figure 6 Expression levels of NK cell receptors on total NK cells and subsets. Percentages of total NK cells **(A)** and NK cell subsets **(B, C)** expressing specific activating (NKG2C, NKG2D) and inhibitory (NKG2A, KIR2DL1, KIR2DL/L3, KIR3DL1) receptors.

Supplementary Table 1 Symptoms experienced after first vaccination.

Supplementary Table 2 Symptoms experienced after second vaccination.
