# Supplementary figures and images for: Natural killer cells and BNT162b2 mRNA vaccine reactogenicity and durability
Source: Front Immunol. 2023 Aug 25;14:1225025. doi: 10.3389/fimmu.2023.1225025 (PMC10497936; doi:10.3389/fimmu.2023.1225025)

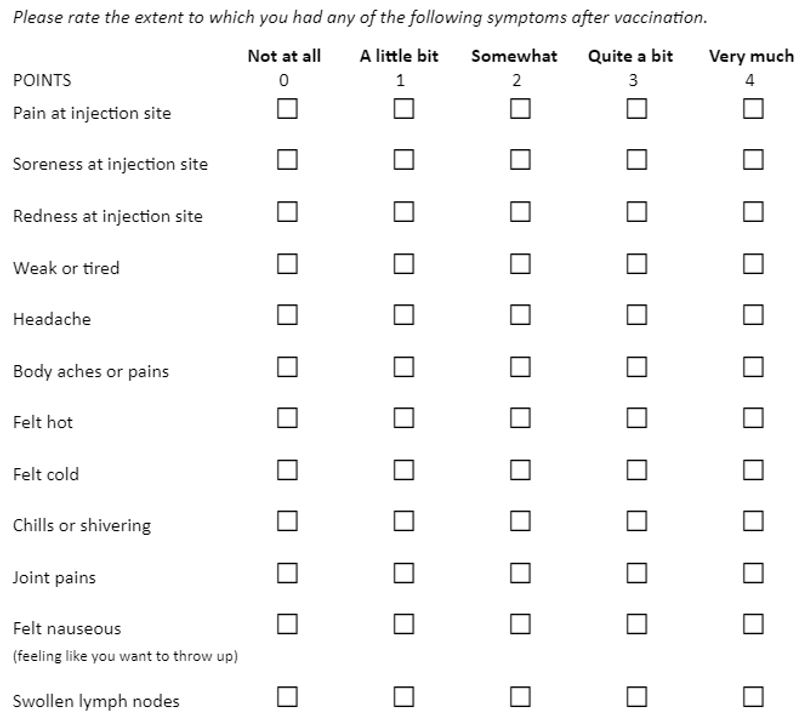

Supplement: Supplementary Figure 1 — Symptoms questionnaire filled out by study participants after each SARS-CoV-2 vaccination. Participants of the PASS study answered these 12 questions following vaccination and selected on a scale from 0-4, where “0” means they did not experience the symptom at all and “4” means they very much so experienced the symptom. The total maximum score possible was 48 and the total lowest score possible was 0. [file DataSheet_1.zip › Supplementary Figure 1.JPEG]

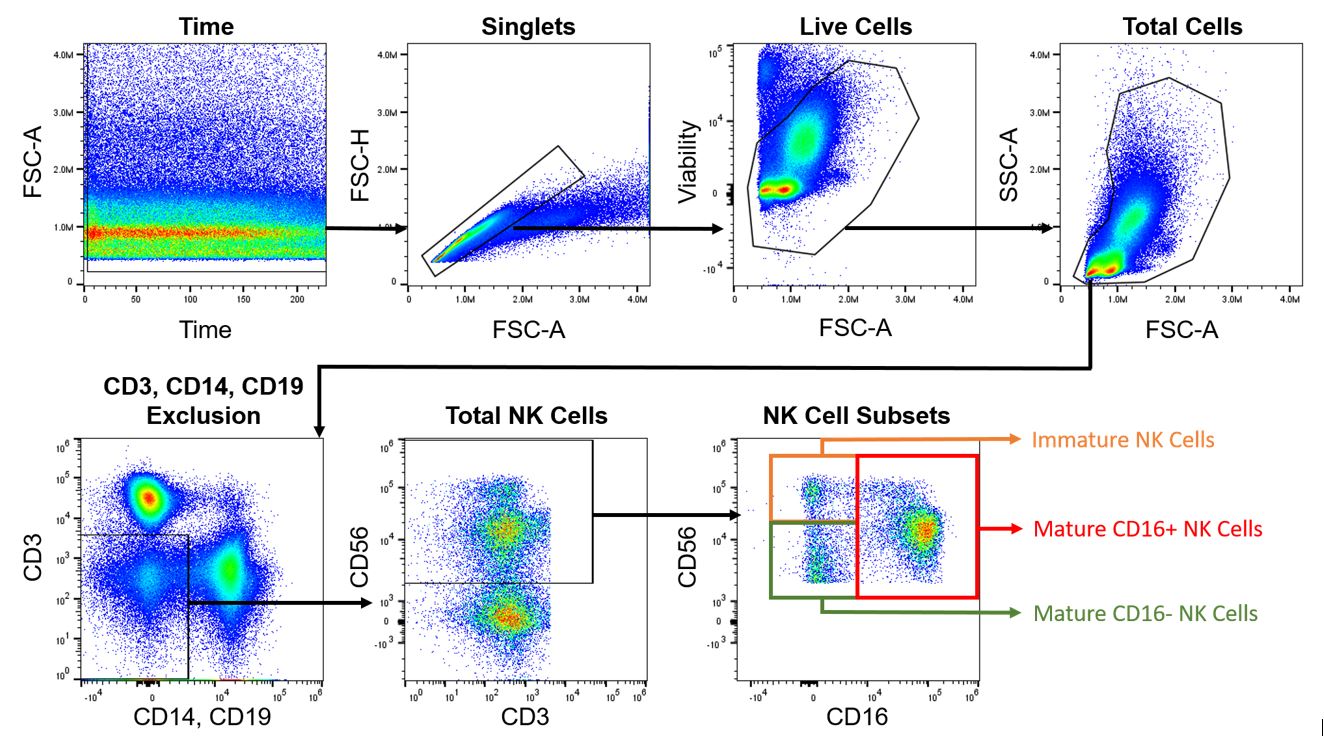

Supplement: Supplementary Figure 1 — Symptoms questionnaire filled out by study participants after each SARS-CoV-2 vaccination. Participants of the PASS study answered these 12 questions following vaccination and selected on a scale from 0-4, where “0” means they did not experience the symptom at all and “4” means they very much so experienced the symptom. The total maximum score possible was 48 and the total lowest score possible was 0. [file DataSheet_1.zip › Supplementary Figure 2.JPEG]

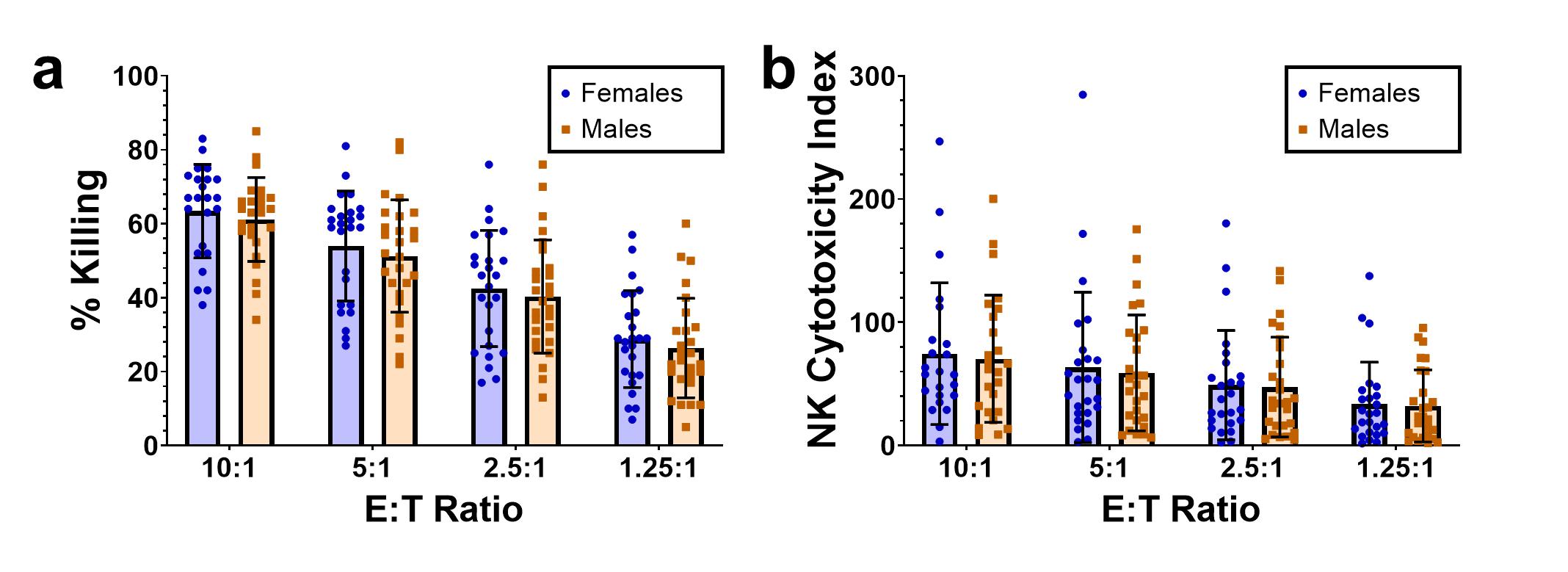

Supplement: Supplementary Figure 1 — Symptoms questionnaire filled out by study participants after each SARS-CoV-2 vaccination. Participants of the PASS study answered these 12 questions following vaccination and selected on a scale from 0-4, where “0” means they did not experience the symptom at all and “4” means they very much so experienced the symptom. The total maximum score possible was 48 and the total lowest score possible was 0. [file DataSheet_1.zip › Supplementary Figure 3.JPEG]

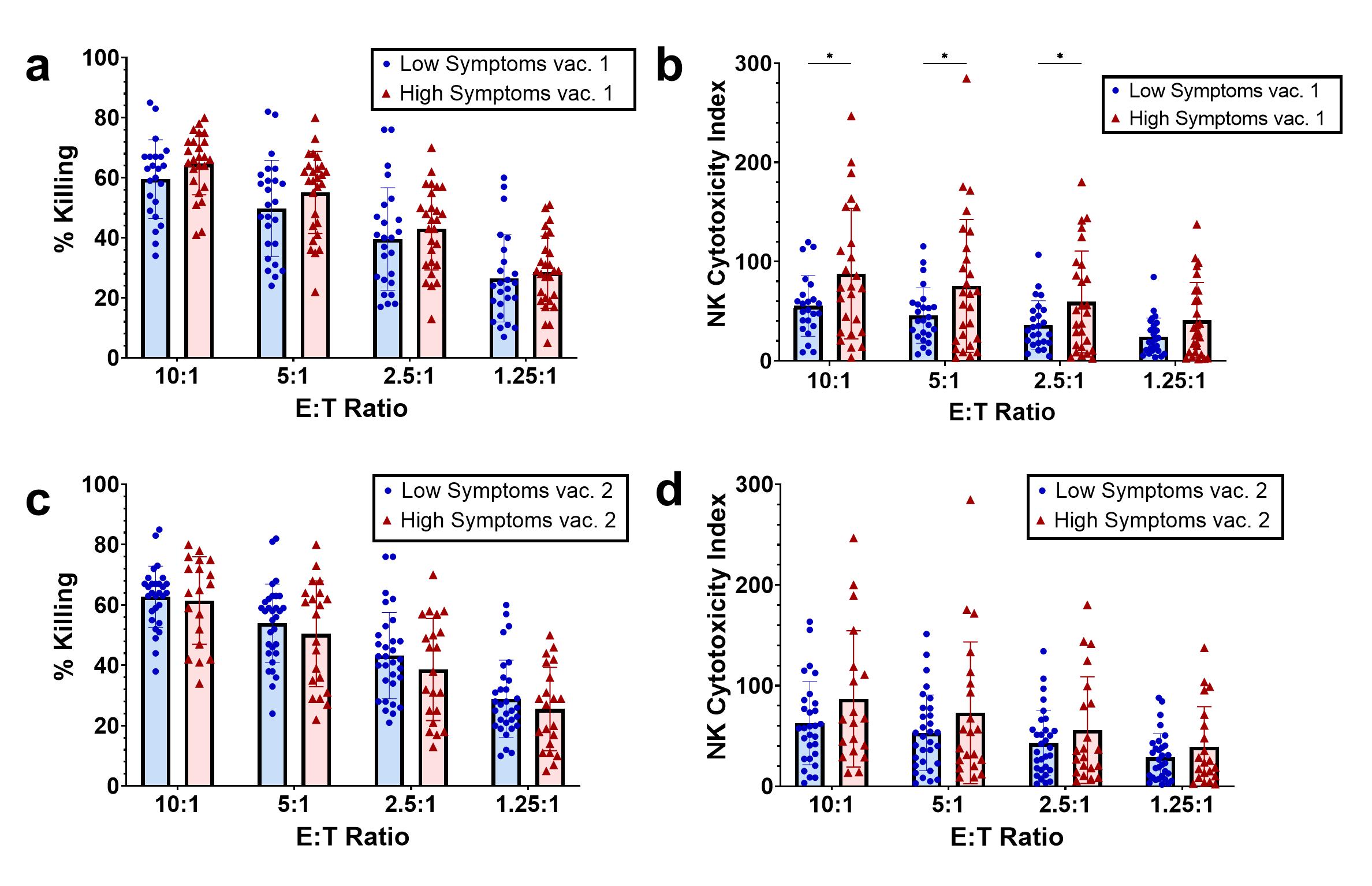

Supplement: Supplementary Figure 1 — Symptoms questionnaire filled out by study participants after each SARS-CoV-2 vaccination. Participants of the PASS study answered these 12 questions following vaccination and selected on a scale from 0-4, where “0” means they did not experience the symptom at all and “4” means they very much so experienced the symptom. The total maximum score possible was 48 and the total lowest score possible was 0. [file DataSheet_1.zip › Supplementary Figure 4.JPEG]

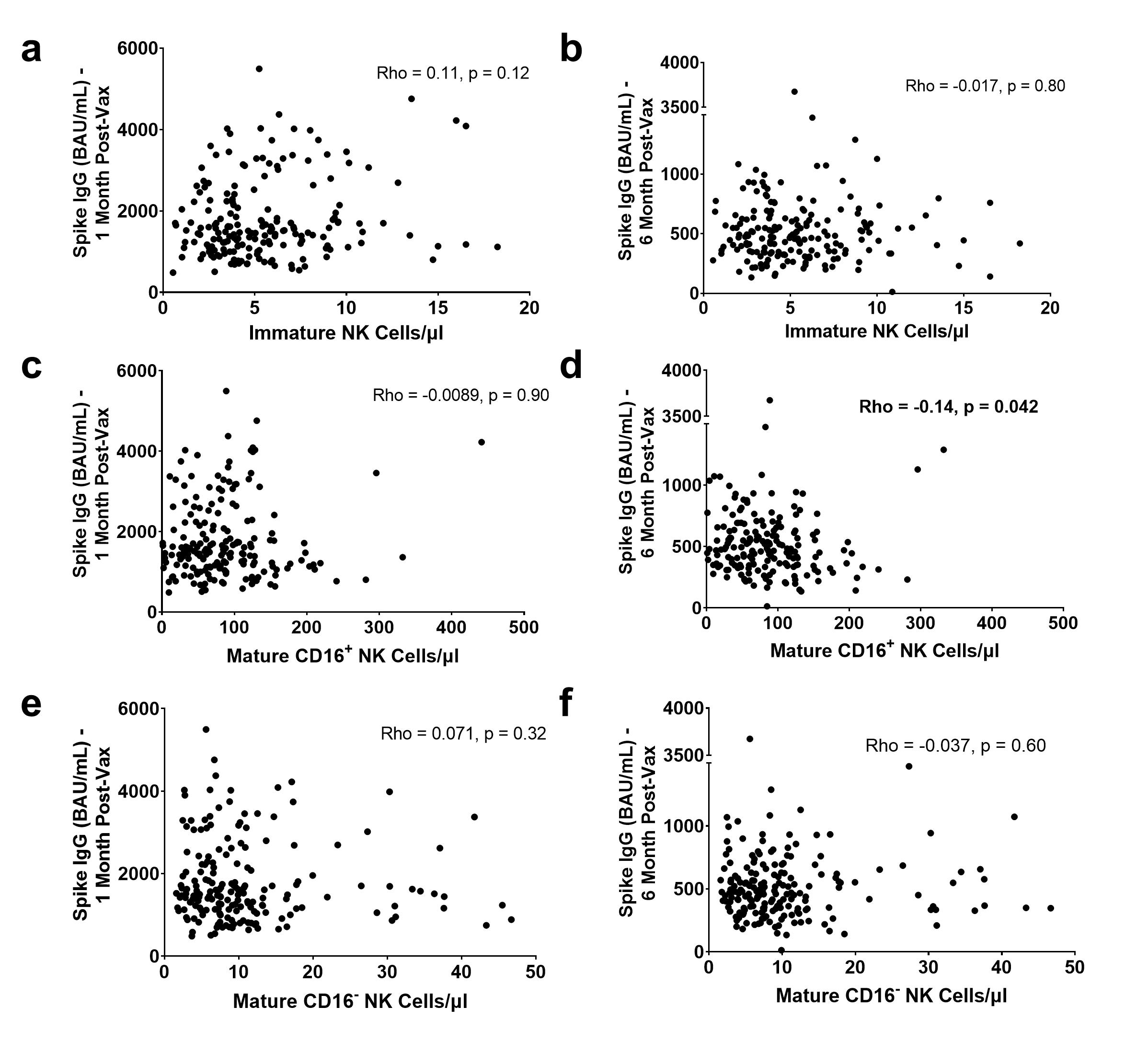

Supplement: Supplementary Figure 1 — Symptoms questionnaire filled out by study participants after each SARS-CoV-2 vaccination. Participants of the PASS study answered these 12 questions following vaccination and selected on a scale from 0-4, where “0” means they did not experience the symptom at all and “4” means they very much so experienced the symptom. The total maximum score possible was 48 and the total lowest score possible was 0. [file DataSheet_1.zip › Supplementary Figure 5.JPEG]

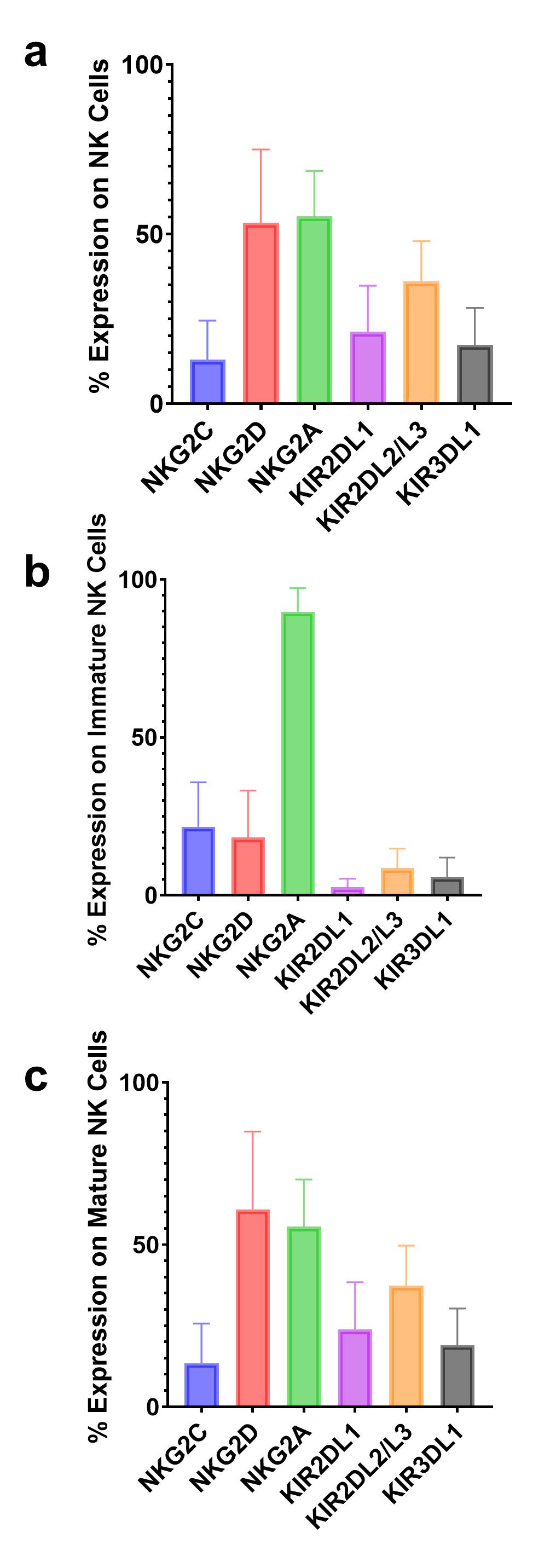

Supplement: Supplementary Figure 1 — Symptoms questionnaire filled out by study participants after each SARS-CoV-2 vaccination. Participants of the PASS study answered these 12 questions following vaccination and selected on a scale from 0-4, where “0” means they did not experience the symptom at all and “4” means they very much so experienced the symptom. The total maximum score possible was 48 and the total lowest score possible was 0. [file DataSheet_1.zip › Supplementary Figure 6.JPEG]

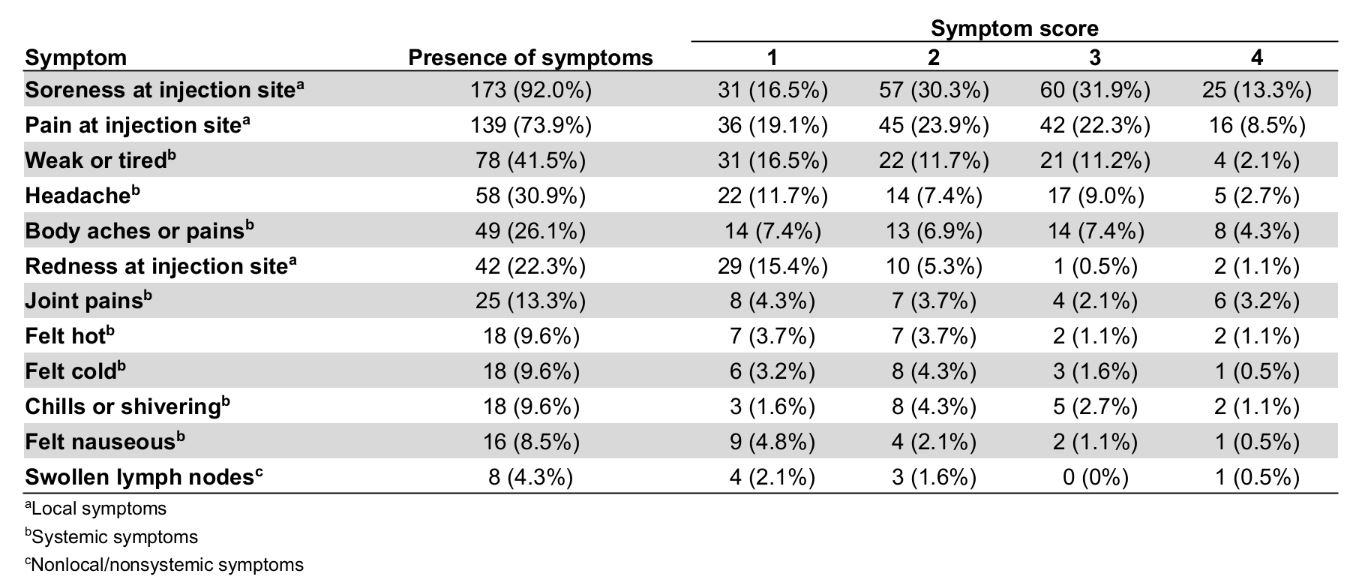

Supplement: Supplementary Figure 1 — Symptoms questionnaire filled out by study participants after each SARS-CoV-2 vaccination. Participants of the PASS study answered these 12 questions following vaccination and selected on a scale from 0-4, where “0” means they did not experience the symptom at all and “4” means they very much so experienced the symptom. The total maximum score possible was 48 and the total lowest score possible was 0. [file DataSheet_1.zip › Supplementary Table 1.JPEG]

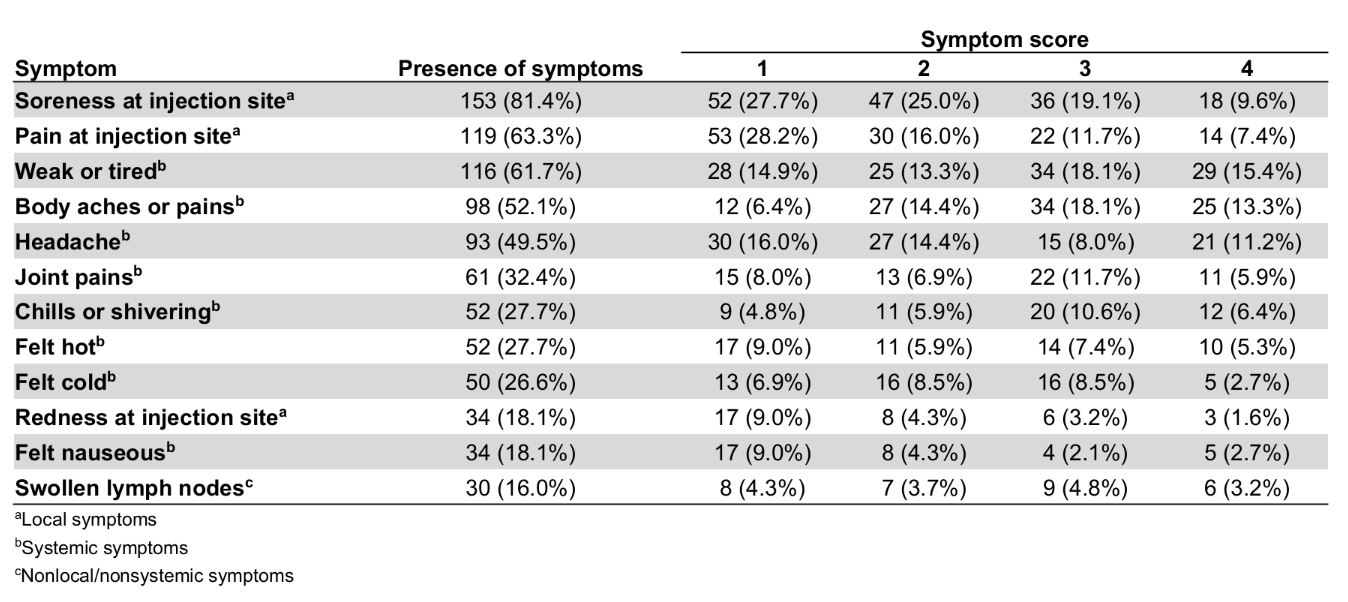

Supplement: Supplementary Figure 1 — Symptoms questionnaire filled out by study participants after each SARS-CoV-2 vaccination. Participants of the PASS study answered these 12 questions following vaccination and selected on a scale from 0-4, where “0” means they did not experience the symptom at all and “4” means they very much so experienced the symptom. The total maximum score possible was 48 and the total lowest score possible was 0. [file DataSheet_1.zip › Supplementary Table 2.JPEG]
